# Effect of low-normal and high-normal IGF-1 levels on memory and wellbeing during growth hormone replacement therapy: a randomized clinical trial in adult growth hormone deficiency

**DOI:** 10.1186/s12955-018-0963-2

**Published:** 2018-07-06

**Authors:** Christa C. van Bunderen, Jan Berend Deijen, Madeleine L. Drent

**Affiliations:** 1 Department of Internal Medicine, section of Endocrinology, Neuroscience Campus Amsterdam, VU University Medical Center, De Boelelaan 1117, 1081 HV Amsterdam, the Netherlands; 2 Department of Clinical Neuropsychology, VU University, Amsterdam, the Netherlands

**Keywords:** Growth hormone deficiency, Growth hormone treatment, Cognition, Memory, Mood, Insulin-like growth factor-1

## Abstract

**Background:**

The aim of the present study was to investigate the effect of low-normal and high-normal levels of IGF-1 in growth hormone (GH) deficient adults on cognition and wellbeing during GH treatment.

**Methods:**

A randomized, open-label, clinical trial including 32 subjects receiving GH therapy for at least 1 year. Subjects were randomized to receive either a decrease (IGF-1 target level of − 2 to − 1 SDS) or an increase of their daily GH dose (IGF-1 target level of 1 to 2 SDS) for a period of 24 weeks. Memory was measured by the Cambridge Neuropsychological Test Automated Battery, selecting the Pattern Recognition Memory task and the Spatial Working Memory. Wellbeing was measured as mood by the Profile of Moods States questionnaire, and quality of life by the Nottingham Health Profile and QoL Assessment in GH Deficiency in Adults questionnaires.

**Results:**

Data from 30 subjects (65.6% male, mean age 46.6 (9.9 SD) years), who fulfilled the target levels, were analyzed. Females in the low dose treatment arm were found to have a better working memory and a better strategic memory control after 24 weeks as opposed to the females in the high treatment arm. With respect to mood, the decrease in IGF-1 levels in females within the low treatment arm was associated with more fatigue and less vigor.

**Conclusions:**

The adjustment of GH dose in female patients seems to have a narrow window. A dose too high may impair prefrontal cognitive functioning, while a dose too low may result in decreased vigor.

**Trial registration:**

This study is registered with ClinicalTrials.gov, number NCT01877512.

## Background

Growth hormone (GH) deficiency in adults may lead to a broad range of detrimental physical and/or psychological effects [1], while there is evidence that GH treatment has a positive effect on body composition, lipid profile, hypertension, bone density, and quality of life (QoL) [2–5]. With respect to psychological functions, GH deficiency has been found to be associated with cognitive deficits. For example, specific cognitive deficits observed are lapses of attention, difficulty in concentrating, forgetfulness, impaired spatial learning and lower perceptual speed [6–9]. Also, GH deficiency is accompanied with subnormal IQ scores, memory impairment, and a low educational level. These manifestations are associated with a low insulin-like growth factor-1 (IGF-1) concentration, suggesting that subnormal cognitive performance is related to GH deficiency [10]. Beyond cognitive impairment, GH deficiency has been found to worsen wellbeing as well. In several studies, GH deficient patients were found to show emotional instability, a lack of energy, difficulties in social and sexual functioning, and to suffer from sleeping problems [9, 10]. In clinical studies, GH replacement therapy appeared to improve the impaired cognitive and wellbeing in GH deficient adults [11–15]. However, in several studies an absence of any GH treatment effect on cognitive functions has also been observed [16–19]. Studies on the effects of GH treatment on wellbeing also show conflicting results. For instance, there are reports that GH therapy improves self-perceived wellbeing, increases energy and decreases pain, anxiety and depression [14, 15, 20–23]. However, no changes in psychological wellbeing or QoL after GH treatment have been found as well [13, 16, 24]. Whether this is tested by generic or disease-specific questionnaire should not be discarded when interpreting these negative results.

Also, a linear association of psychological impairment with circulating IGF-1 level is demonstrated. Indeed, higher IGF-1 levels in GH deficient patients and healthy subjects have been found to be associated with a better cognitive functioning [25–27]. With respect to mood, higher IGF-1 levels in adults with GH deficiency were found to be associated with less depression, fatigue, tension, anxiety and with more vigor [28, 29].

The effect of GH and IGF-1 on cognitive functioning and wellbeing may be mediated through mechanisms involving the stimulation of the GH and IGF-1 receptors in the brain. GH and IGF-1 can pass the blood-brain barrier and there are binding-sites for GH and IGF-1 in discrete regions of the brain. GH binding sites have been demonstrated in the choroid plexus, hypothalamus, hippocampus and frontal cortex [30, 31]. Also for IGF-1 there are specific binding sites identified, such as in the choroid plexus, frontal cortex, putamen, hippocampus, cerebellum, amygdala, thalamus and substantia nigra [32, 33]. Currently, there is substantial evidence that IGF-1 is involved in neuroprotection, regeneration and brain plasticity [34]. Anabolic hormones may also have direct effect on cells producing ß-endorphin and this could be one of the mechanisms behind the improvement of wellbeing seen during GH treatment [35].

The above cited studies did not distinguish between males and females. However, it may be important to take gender differences into account. Although healthy females secrete 2–3 fold greater amounts of GH than males their IGF-1 levels are similar to those of males [36]. As the cognitive effects of GH treatment may be dependent on the levels of both GH and IGF-1 obtained by GH treatment, cognitive effects may be expected to be different in males and females.

The aim of the present study was to investigate the effect of low-normal and high-normal levels of IGF-1 in GH deficient adults on cognition and wellbeing during GH treatment. We hypothesized that higher levels of IGF-1 would improve memory and wellbeing, with different effects in females than in males.

## Methods

### Study design

This study presents outcome data on cognition and wellbeing from a randomized, open-label, clinical trial conducted at one university hospital (VU University Medical Center, Amsterdam, The Netherlands) which compared de- and increasing GH dose for 24 weeks with low-normal and high-normal IGF-1 target levels for efficacy and safety measures of GH replacement therapy [37]. At entry, subjects were receiving GH treatment according to general clinical practice (daily subcutaneous injections of somatropin using automated pen systems). Subjects were selected on having an IGF-1 concentration between − 1 and 1 standard deviation score (SDS) during GH replacement therapy. Randomization was done by a computer-generated random sequence and was stratified by gender. Subjects were randomized to receive either a decrease of their regular dose of GH treatment (IGF-1 target level of − 2 to − 1 SDS) (low dose = LD group), or an increase of their regular dose (IGF-1 target level of 1 to 2 SDS) (high dose = HD group), for 24 weeks. After 4 weeks adjustment of GH dose was initiated when the target level of IGF-1 was not reached. At visit one (baseline) and visit two (after 24 weeks) blood samples were drawn and measurements were performed to assess cognition and wellbeing.

### Patients

The study group consisted of 32 adult patients with documented severe GH deficiency and more than 1 year of GH treatment, with an IGF-1 level between − 1 and 1 SD score (SDS), stable for at least 6 months. Other pituitary hormone deficiencies had to be substituted when indicated and be stable for at least 6 months and during follow up. Severe GH deficiency was diagnosed prior to the study and defined according to the consensus guidelines of the GH Research Society for the diagnosis and treatment of adults with GH deficiency [38]. Patients were not eligible if they had a recent or current malignancy, craniopharyngioma as cause of hypopituitarism, were (planning on becoming) pregnant, or had a cardiovascular event within 1 year before recruitment. Patients were included after oral information and signed informed consent. The study protocol was approved by the Ethics Committee of the VU University Medical Center, Amsterdam. The study was performed according to Good Clinical Practice and the Declaration of Helsinki. This study is registered with ClinicalTrials.gov, number NCT01877512 [37].

### Biochemical methods

Blood samples were drawn after an overnight fast prior to every visit. Total IGF-1 was measured by a non-competitive (sandwich), chemiluminescence immunoassay (Liaison, DiaSorin S.p.A., Italy).

### Memory

Cognition was tested by two tasks for visual memory and executive function using the Cambridge Neuropsychological Test Automated Battery (CANTAB) [39]. Subjects were tested individually in a sound attenuated room by the same investigator at the same time period of the day. The whole test procedure took about 30 min. The CANTAB tests were conducted using a 17" ELO touch screen. A short motor screening task was performed to ensure participants were unimpaired in their ability to respond to the stimuli, and to familiarize them with the computerized procedure. The performance on the following tests was evaluated: 1) the Pattern Recognition Memory (PRM) task to investigate visual pattern recognition memory in a 2-choice forced discrimination paradigm. The PRM task involves temporal lobe function [40]. The outcome measure is the percentage of correctly recalled visual patterns. 2) The Spatial Working Memory (SWM) task to investigate executive function, working memory, and planning. As these functions are all associated with the frontal area of the brain the SWM is considered to involve frontal lobe function [41, 42]. The selected outcome measures were the number of errors made during the test (total errors) and a score for the use of a strategy. A high strategy score represents poor strategy use [43].

### Wellbeing

To assess mood the Profile of Moods States (POMS) questionnaire was administered. A shortened Dutch version of 32 items was used for measuring depression, anger, fatigue, tension, and vigor [44]. The POMS answers are graded on a 5-point scale ranging from ‘not at all’ (scale 0) to ‘extremely’ (scale 4). Higher scores for depression (scores 0–32), anger (scores 0–28), fatigue (scores 0–24) and tension (scores 0–24) reflect a negative mood; higher scores for vigor (scores 0–20) reflect a better mood. QoL was assessed by using two different questionnaires, one disease-specific and one general. The disease-specific questionnaire was the Dutch version of the QoL Assessment of GH deficiency in Adults (QoL-AGHDA) including 25 questions. The general questionnaire was the Nottingham Health Profile (NHP), a frequently used health status instrument with 38 dichotomous items that measures physical, emotional, and social distress. It yields an overall score and sub-section scores (physical mobility, energy, pain, emotional reactions, sleep, and social isolation). High scores indicate a poor QoL.

### Statistical analyses

Categorical baseline data are expressed as percentage and continuous data as mean (SD). Categorical data were analyzed by means of chi square tests and continuous data by means of independent *t*-tests. For between-group differences for change over time General Linear Model for repeated measures was used with Group (HD versus LD) and Gender as between subjects factor, and Measurement (baseline versus week 24) as repeated measures factor. If an interaction between Group, Measurement and Gender was observed, separate ANOVAs per gender with Group as between subjects factor and Measurement as repeated measures factor were performed. For the different outcome measures baseline values served as covariates to adjust for regression to the mean. Moreover, at baseline the LD and HD male groups differed with respect to childhood onset (CO) and adult onset (AO) GH deficiency and IGF-1 SDS. As a *t*-test indicated that the mean IGF-1 SDS in the CO group was significant higher than that in the AO group (mean IGF-1 SDS in CO: 0.24 and in AO: − 0.47, *t*(15) = 3.20, *p* = 0.006), IGF-1 SDS was also used as covariate. Two sided *P* values 0.05 or less were considered significant. In case of hypotheses with expected results in one direction one-tailed *t*-tests (which is indicated in the text) were used. Statistical analyses were performed by the statistical software package IBM SPSS statistics 20.0 (SPSS Inc., Chicago, IL).

## Results

### Baseline characteristics

Between May 31, 2013, and April 11, 2014, we enrolled 32 patients. An invitation was send to 92 eligible patients. Reasons for not participating were lack of time, travel distance, or reluctance to risk deterioration. Table 1 shows the baseline characteristics of the study groups stratified by gender. The groups were mostly comparable, except for two significant differences. First, the presence of males with CO GH deficiency in the LD group was higher than that in the HD group. Second, the IGF-1 SDS of the males was higher in the LD group than in the HD group. Most prevalent underlying diagnosis of GH deficiency was a pituitary tumor. Off all 13 pituitary tumors, six were a non-secreting adenoma, five a prolactinoma, and two were an ACTH producing adenoma. Other etiologies included radiotherapy for other brain tumors, pituitary apoplexia, empty sella, head trauma, idiopathic, or congenital GH deficiency. The medical history (for CVD and diabetes mellitus), smoking habits and alcohol use was similar between all groups.

**Table 1:** Baseline characteristics for males and females of the low dose group (IGF-1 target level between − 2 and − 1 SDS) and the high dose group (IGF-1 target level between 1 and 2 SDS)

	Males		Females	
	Low Dose *n* = 10	High Dose *n* = 11	Low Dose *n* = 6	High Dose *n* = 5
Age (years)	46.3 (11.2)	47.4 (8.9)	49.1 (10.7)	44.3 (10.9)
CO GH deficiency (%)	80	27*	33	0
Duration GH treatment (years)	18.6 (9.4)	13.4 (6.3)	8.9 (5.3)	4.8 (1.8)
IGF-1 in SDS	0.29 (0.62)	−0.38 (0.38)**	−0.08 (0.57)	− 0.03 (0.42)
BMI (kg/m^2^)	25.6 (3.4)	28.6 (3.8)	33 (14.5)	29 (5.2)
Cranial radiotherapy (%)	10	18	0	0
Pituitary surgery (%)	10	54	17	40
Isolated GH deficiency (%)	20	26	33	0
LH/FSH deficiency (%)	80	45	33	40
TSH deficiency (%)	70	64	17	80
ACTH deficiency (%)	80	64	33	60
ADH deficiency (%)	0	36	0	0
Diabetes mellitus (%)	10	0	33	0
CVD (%)	0	18	50	40
Married (%)	60	73	83	60
Education 10–13 years (%)	30	18	16	40
Education > 13 years (%)	70	82	83	60

Values are mean (SD) unless stated otherwise

*CO* childhood onset GH deficiency, *GH* growth hormone, *IGF-1 in SDS* insulin like growth factor-1 in standard deviation score, *BMI* body mass index, *LH/FSH* luteinising hormone/follicle stimulating hormone, *TSH* thyroid stimulating hormone, *ACTH* adrenocorticotropic hormone, *ADH* antidiuretic hormone, *CVD* cardiovascular disease

*p < 0.05, ** *p* < 0.01 (low dose versus high dose)

### Follow up

**Fig. 1:**
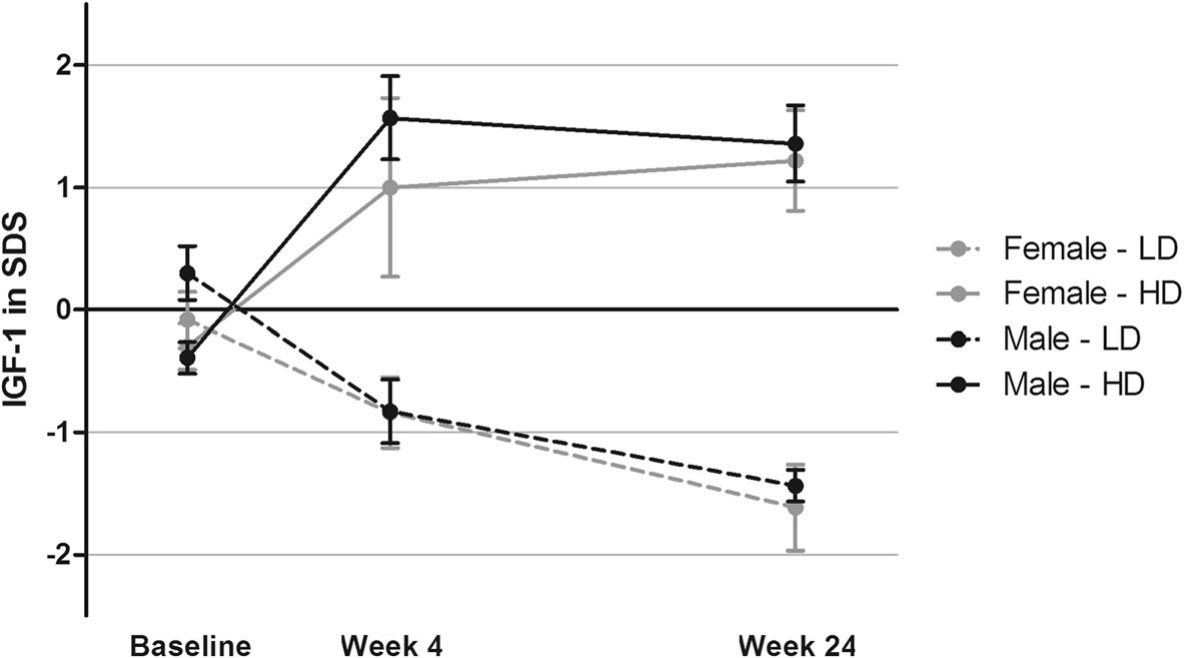
Mean serum total IGF-1 level in SD score (± SEM) at baseline, after 4 weeks of GH treatment and at end of follow up in the low dose (LD) and high dose (HD) female and male group

After start of the study one subject withdrew due to personal reasons. One subject was excluded from the analyses due to the inability to reach the proper IGF-1 target level. The final analyses were conducted with 15 subjects in the LD group and 15 subjects in the HD group. In males, the IGF-1 concentration decreased from 23.11 (SD 5.18) at baseline to 12.98 (SD 2.0) nmol/L (*p* < 0.001) after 24 weeks in the LD group, and increased from 18.40 (SD 3.31) to 28.2 (SD 5.27) nmol/L (*p* = 0.002) in the HD group. In females, the IGF-1 concentration decreased from 18.83 (SD 3.19) at baseline to 11.62 (SD 2.56) nmol/L (*p* = 0.02) after 24 weeks in the LD group, and increased from 18.80 (SD 1.48) to 28.0 (SD 5.52) nmol/L (*p* = 0.01) in the HD group. Figure 1 shows the IGF-1 levels in standard deviation scores (SDS) during follow up for both treatment regimes stratified by gender. Table 2 shows the different GH doses stratified by gender before and after follow up.

**Table 2:** Different GH doses stratified by gender at baseline and after follow up

		Low Dose		High Dose	
GH dose at baseline (mg/day)	Male	0.20	(0.25)	0.13	(0.23)
	female	0.38	(0.39)	0.50	(0.30)
GH dose at week 24 (mg/day)	Male	0.08	(0.06)	0.40	(0.28)
	female	0.15	(0.21)	1.00	(0.83)

Values are median (interquartile range)

### Memory

**Fig. 2:**
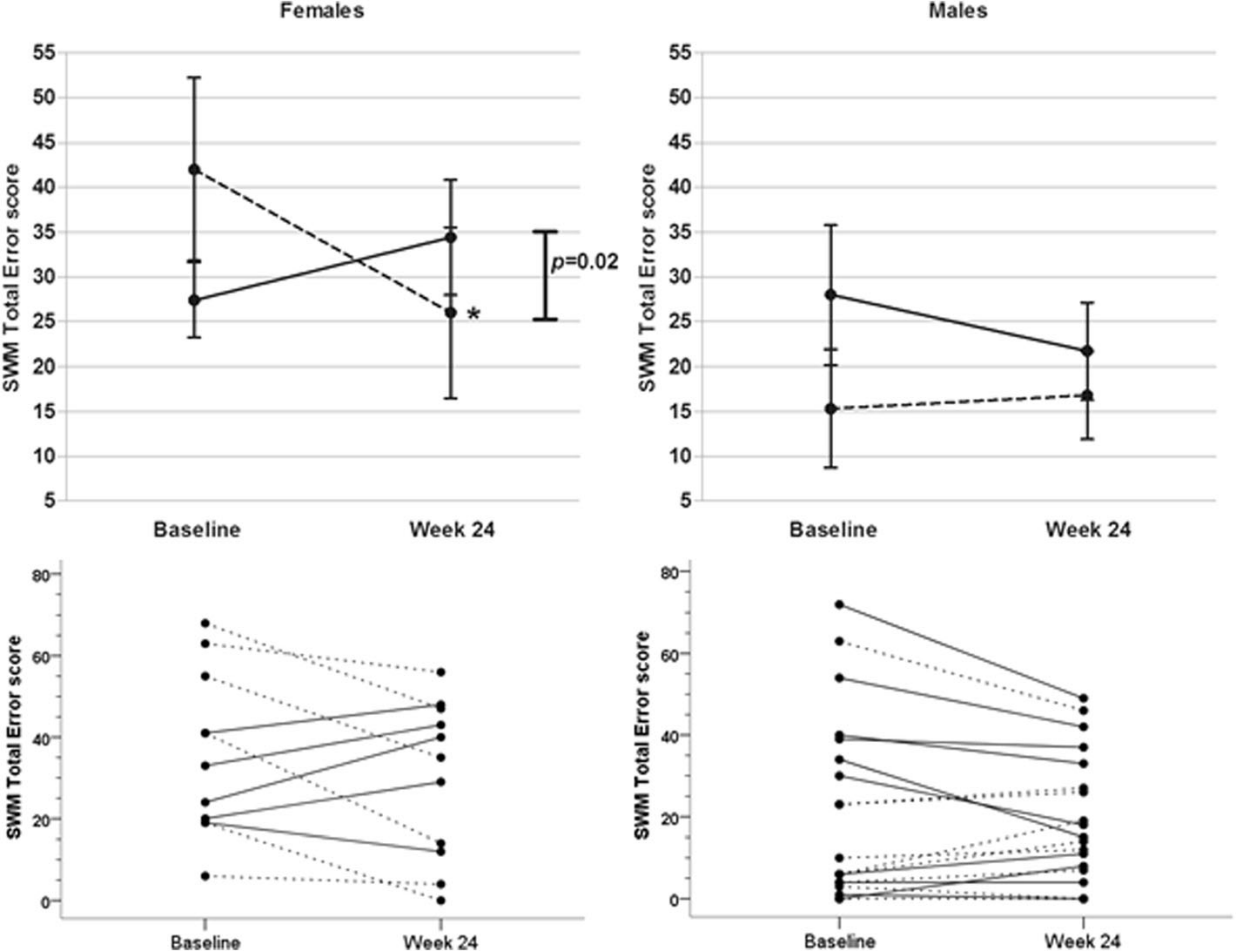
Mean (± SEM) and individual SWM Total Error scores of females and males in the low dose (dashed line) and high dose (solid line) groups at baseline and at week 24 (lower scores meaning better performance). * *p* < 0.05 for mean score at week 24 compared to baseline per group

With respect to SWM Total Errors, there was a significant interaction for Measurement x Group x Gender, *F*(1,26) = 19.23, *p <* 0.001, *partial* η^2^ = 0.42. Analyses performed separately for each gender with SWM Total Errors score and IGF-1 SDS at baseline as covariate, revealed in the female group a significant interaction between Group and Measurement, *F*(1,7) = 10.99, *p* = 0.02, *partial* η^2^ = 0.61. Post hoc *t*-tests indicated a significant lower SWM Total Errors score of the females in the LD group at week 24 as compared to baseline, *t*(5) = 4.14, *p* = 0.009. No significant effect was found in males. This result indicates that females in the LD group perform better on the SWM task after 24 weeks of treatment compared to the HD group, and compared to baseline (Fig. 2).

With respect to SWM Strategy, there was a significant interaction for Measurement x Group x Gender, *F*(1,26) = 4,22, *p* = 0.05, *partial* η^2^ = 0.14. Analyses performed separately for each gender with SWM Strategy score and IGF-1 SDS at baseline as covariate, revealed in the female group, but not in the male group, a marginally significant interaction between Group and Measurement, *F*(1,7) = 5.16, *p* = 0.057, *partial* η^2^ = 0.42. Post hoc *t*-tests did not indicate a significant lower SWM Strategy score of the females in the LD nor in the HD group at week 24 as compared to baseline (*p* > 0.05). This result indicates that females in the LD group perform better on the SWM task after 24 weeks of treatment compared to the HD group.

With respect to the PRM test scores no significant results were found. All means (SD) of the memory tests are summarized in Table 3.

**Table 3:** Mean (SD) of cognitive tests and mood scales in males and females at baseline and week 24 for the low and high dose group

	Males				Females			
	Low Dose *n* = 9		High Dose *n* = 10		Low Dose *n* = 6		High Dose *n* = 5	
	Baseline	Week 24	Baseline	Week 24	Baseline	Week 24	Baseline	Week 24
MEMORY								
PRM (%)	91.2 (8.7)	92.6 (5.8)	85.8 (15.2)	91.2 (8.2)	88.9 (8.2)	90.3 (10.1)	91.7 (8.3)	91.7 (11.4)
SWM TE	15.3 (19.7)	16.8 (14.7)	28 (24.7)	21.7 (17.2)	42 (24.9)	26(23.3)**	27.4 (9.4)	34.4 (14.3)
SWM STR	38.3 (11.1)	36.3 (8.8)	43.3 (9)	40.1(7.8)**	44.3 (6.4)	40.8 (9.4)	43.4 (2.1)	46 (3.5)
MOOD								
Depression	2.9 (3.2)	4.1 (6.7)	2.6 (3.3)	1.2 (2.5)	4.3 (4.9)	7 (7.3)	5.2 (3.1)	5 (6)
Anger	5.4 (6.2)	4.9 (5.4)	4.8 (4.7)	4.2 (4.2)	9 (7.7)	7 (5.8)	5.2 (5.7)	5.6 (7.2)
Fatigue	5.4 (6)	5.3 (6.6)	3.9 (3.4)	3 (1.8)	9.8 (7.5)	13.2 (3.7)	4.4 (4.7)	3.6 (3.8)
Tension	3 (2.9)	3.7 (4)	4.5 (4.6)	3.7 (3.6)	4.5 (4.6)	7.8 (5.3)	6.2 (5.5)	5 (5.4)*
Vigor	11.3 (4.1)	12.8 (4.8)	12.5 (3.4)	11.8 (3.9)	11.5 (5.2)	7.8 (4)*	12.8 (1.5)	12 (2.7)

PRM (%) = Pattern Recognition Memory (percent correct)

*SWM TE* Spatial Working Memory Total Errors, *SWM STR* Spatial Working Memory Strategy

* *p* < 0.05, ** *p ≤* 0.01; week 24 versus baseline

### Wellbeing

With respect to mood scores for Anger, Depression and Tension no significant interaction for Measurement x Group x Gender and Measurement x Group per gender was found. All means (SD) of the mood scales are shown in Table 3.

**Fig. 3:**
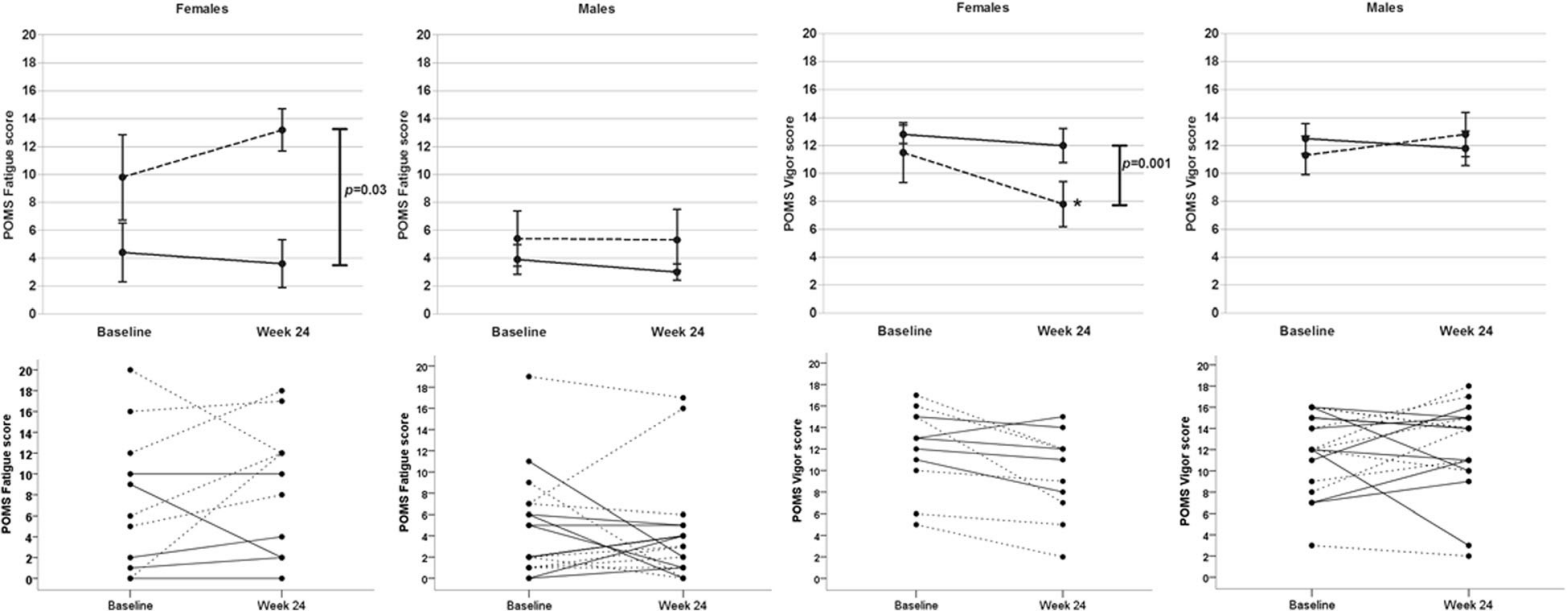
Mean (± SEM) and individual POMS Fatigue and Vigor scores of females and males in the low dose (dashed line) and high dose (solid line) groups at baseline and at week 24 (higher scores meaning more fatigue and more vigor). * *p* < 0.05 for mean score at week 24 compared to baseline per group

With respect to Fatigue, there was a significant interaction for Measurement x Group x Gender with Fatigue score and IGF-1 SDS at baseline as covariates, *F*(1,24) = 5.97, *p* = 0.02, *partial* η^2^ = 0.20. ANOVAs separately performed for males and females with Fatigue score and IGF-1 SDS at baseline as covariates yielded no significant interaction effect in males between Group and Measurement on Fatigue (*p* > 0.05). In females, a significant interaction was found between Group and Measurement, *F*(1,7) = 7.9, *p* = 0.03, *partial* η^2^ = 0.53. Post hoc *t*-tests did not indicate any significant difference in Fatigue score of the females in the LD or HD group at week 24 as compared to baseline (*p* > 0.05). Thus, females within the LD group show a higher increase in Fatigue after 24 weeks of treatment relative to females in the HD group (Fig. 3).

With respect to Vigor, the interaction for Measurement x Group x Gender approached significance, *F*(1,26) = 3.79, *p* = 0.06, *partial* η^2^ = 0.13. Therefore analyses were performed separately for males and females with Vigor score and IGF-1 SDS at baseline as covariates. With respect to males no significant interaction effect between Group and Measurement was found (*p* > 0.05). In females, a significant interaction was found between Group and Measurement, *F*(1,7) = 29.8, *p* = 0.001, *partial* η^2^ = 0.81. Post hoc *t*-tests indicated a significant lower Vigor score of the females in the LD group at week 24 as compared to baseline, *t*(5) = 3.38, *p* = 0.02. Thus, females within the LD group show a reduced Vigor after 24 weeks of treatment compared to the HD group, and compared to baseline (Fig. 3).

With respect to QoL outcome scores no significant interaction for Measurement x Group x Gender and Measurement x Group per gender was found.

## Discussion

The present study demonstrated that, with respect to cognitive functioning as well as to wellbeing, gender appeared to render different effects of low and high dose GH treatment. Notably, these effects were not observed in all cognitive domains studied.

The PRM subtest is a visual recognition memory task relying on medial temporal lobe functioning. In contrast, the SWM task establishes working memory and strategic memory control, which functions rely on frontal lobe functioning. In the present study no differential effects of the low and high dose GH treatment were found in males nor in females, with respect to PRM. Therefore, it may be concluded that the change in GH treatment did not specifically affect medial temporal lobe functioning. In contrast, SWM was affected by the different GH doses, but only in females. In males the low and high dose groups did not have a different effect on SWM, while in females a clear distinction could be made between the effects of the low and the high dose group. Females in the LD group were found to have a better working memory after 24 weeks as opposed to the females in the HD group. In addition, the females in the LD group showed better strategic memory control after 24 weeks, as opposed to the females in the HD group. These results clearly indicate that in females cognitive performance relying on frontal lobe functioning benefits from a LD GH treatment, but not from an increased GH dose. At the moment that the GH dose was decreased or increased the GH-deficient patients in the present study were already treated with GH for more than 1 year (and IGF-1 levels being between − 1 and 1 SDS for at least 6 months). As a decrease of the previously given dose benefits the cognitive functioning in females, the original dose may have also been too high to be optimal for cognitive function, in particular frontal lobe mediated memory processes. It may well be true that the IGF-1 levels obtained during long-term GH administration are too high to preserve or improve memory functions, and may even result in memory impairment. In a former study on the cognitive effects of GH treatment in adult survivors of childhood leukaemia, the increase in IGF-1 during the first treatment year was accompanied by a decrease in short-term memory performance. Notably, the decrease in IGF-1 in the second treatment year was accompanied by an improvement of memory performance. The authors conclude that if the increase in GH-induced IGF-1 levels is too high memory functions may be impaired, whereas this memory impairment may be halted when IGF-1 levels are decreased [45]. The results of the present study in females seem to have some similarity with those findings. Thus, the chronically given GH dose may have impaired memory functions, while reducing the dose counteracted the harmful effects on memory function. The finding that specifically frontal memory function is affected may be explained by different IGF-1 receptor densities in temporal and frontal brain area. The highest densities of IGF-1 receptors have been found in the hippocampus, amygdala and parahippocampal gyrus, while intermediate densities were observed in the cerebral cortex [32]. Thus, the lower amount of IGF-1 receptors in the frontal cortex compared to that of IGF-1 receptors in the temporal area may account for a different effect of reduced IGF-1 levels. It may be that higher levels of IGF-1 result in downregulation, and lower levels of IGF-1 in upregulation of IGF-1 receptors in the frontal cortex. The latter leads to a more sensitive neuronal system which may account for better cognitive function. The density of IGF-1 receptors in the temporal lobe may be that high, that the sensitivity of the neuronal system has already reached its upper limit. However, more basic research is needed to verify this theory.

With respect to wellbeing, and in particular to mood, the findings were opposite to those concerning cognition. Females in the LD group were found to have a reduced vigor and increased fatigue after 24 weeks as opposed to the females in the HD group. In males no differences were found. It may thus be concluded that the decrease of GH dose is detrimental for the subjectively perceived vitality. Vigor has been found to be quite closely associated with IGF-1 levels [28, 29]. The present finding that the decrease in IGF-1 levels is associated with more fatigue and less vigor is in line with these former findings. However, we observed these negative effects of decreased IGF-1 levels on these mood states only in females. One explanation for the lack of effect in the male patients could be the adaptation phenomenon observed in GH deficiency, since CO GH deficiency was more prevalent in the male group. Patients with GH deficiency since childhood might not experience the effect of changing GH dose on mood and QoL as patients with adult onset GH deficiency might since the latter have experienced a better mood before. Also, because interrelations between sleep and GH regulation are well documented [46, 47], the low energy and fatigue frequently seen in subjects with GH deficiency could partly reflect alterations of sleep quality. Women report a poorer sleep quality than men across a wide age range [48]. The perception of poorer sleep quality in women may be influenced by affective disorders, which are more common in women and may contribute to a higher incidence of insomnia. Women are at a 40% greater risk for developing insomnia, and the risk ratio grows with age [49]. Indeed, in the present study females reported a larger percentage of sleep problems (NHP) at baseline than males (36% versus 11%, *p* = 0.04, one-tailed). Moreover, the percentage of reported sleep problems was larger compared to baseline in females in LD group (37% versus 27%, *p* = 0.04, one-tailed). Because especially women appear to suffer from sleep disturbances, their sleep quality may benefit most by GH treatment and be most impaired by lowering the GH dose. All together, the reduced vigor may be the consequence of an increase in sleep disturbances in particularly females in the LD group. In the present study no differential effects of the low and high dose GH treatment were found in males nor in females, with respect to QoL scores. A beneficial effect of GH treatment on QoL has been demonstrated in several (mainly long duration) studies reviewed by Hazem et al. [50]. Follow up duration and sample size of the present study might have been insufficient to monitor significant differential effects.

The strength of the present study is that the effect of changing the GH dose was examined concerning memory and wellbeing, separately in females and males. In addition, the effect sizes of the results for memory and vigor appeared to be quite high, indicating that the effects of lowering the GH dose are quite substantial and may be assumed to have clinical significance. A limitation of the study is that the distinction between males and females resulted in quite small sample sizes, with a relative heterogeneous aspect. Therefore, it may be possible that more subtle differences in memory or wellbeing in the low and high dose group could not be observed. Next to the sample size, an important limitation is the open-label design, in particular when investigating wellbeing. We recommend that in future research the effects of GH treatment on memory and wellbeing will be studied in larger samples of male and female patient groups in blinded randomized clinical trials.

## Conclusions

The present results indicate that changing the regular GH dose may have a considerable impact on psychological functioning of female patients with GH deficiency. As could be expected, lowering the dose can result in decreased vigor, which may be associated with a deteriorated sleep quality. Remarkably, a lower dose in females seems to improve prefrontal memory functions. This could be the consequence of a maintenance dose too high for optimal cognitive functioning. Taking these differential effects on cognition and mood into account, the adjustment of GH dose in female patients seems to have a narrow window. A dose too high may impair prefrontal cognitive functioning, while a dose too low may result in decreased vigor. Thus, the present results suggest that a “fine-tuned” intermediate dose may be the best option for maintaining an optimal mental status in female patients.
